# Can exercise training enhance the repeated remote ischaemic preconditioning stimulus on peripheral and cerebrovascular function in high-risk individuals?

**DOI:** 10.1007/s00421-020-04580-6

**Published:** 2021-01-28

**Authors:** Joseph D. Maxwell, Madeleine France, Lucy E. M. Finnigan, Howard H. Carter, Dick H. J. Thijssen, Helen Jones

**Affiliations:** 1 Research Institute of Sports and Exercise Science, Liverpool John Moores University, Tom Reilly Building, Byrom Street, Liverpool, L3 3AF UK; 2 Department of Nutrition, Exercise and Sports, Integrative Physiology Group, University of Copenhagen, Copenhagen, Denmark; 3 Department of Physiology, Radboud Institute of Health Sciences, Radboud University Medical Center, Nijmegen, The Netherlands

**Keywords:** Remote ischaemic preconditioning, Exercise, Cerebral autoregulation, Cardiovascular disease, Vascular function

## Abstract

**Background:**

Repeated exposure to remote ischaemic preconditioning (rIPC; short bouts of non-lethal ischaemia) enhances peripheral vascular function within 1 week; whereas, longer periods of rIPC (~ 1 year) may improve cerebral perfusion. Increasing the ‘dose’ of rIPC may lead to superior effects. Given the similarities between exercise and rIPC, we examined whether adding exercise to the rIPC stimulus leads to greater adaptation in systemic vascular function.

**Methods:**

Nineteen individuals with increased risk for cardiovascular disease (CVD) were randomly allocated to either 8 weeks of rIPC (*n* = 9) or 8 weeks of rIPC + exercise (rIPC + Ex) (*n* = 10). rIPC was applied three times per week in both conditions, and exercise consisted of 50 min (70% heart rate max) of cycling 3 times per week. Peripheral endothelial function was assessed using flow-mediated dilation (FMD) before and after ischaemia–reperfusion (IR). Cerebrovascular function was assessed by dynamic cerebral autoregulation (dCA) and cerebrovascular reactivity (CVR), and cardio-respiratory fitness (*V*O_2peak_) using a maximal aerobic capacity test.

**Results:**

FMD% increased by 1.6% (95% CI, 0.4, 2.8) following rIPC + Ex and by 0.3% (− 1.1, 1.5) in the only rIPC but this did not reach statistical significance (*P* = 0.65). Neither intervention evoked a change in dCA or in CVR (*P* > 0.05). *V*O_2peak_ increased by 2.8 ml/kg/min (1.7, 3.9) following the rIPC + Ex and by 0.1 ml/kg/min (− 1.0, 1.4) following the rIPC only intervention (*P* = 0.69).

**Conclusion:**

Combining exercise with rIPC across an 8-week intervention does not lead to superior effects in cerebrovascular and peripheral vascular function compared to a repeated rIPC intervention in individuals at risk of CVD.

## Introduction

Remote ischaemic preconditioning (rIPC) is characterised by brief benign periods of ischaemia followed by reperfusion which confers protection against ischaemic injury in remote tissue and organs (Przyklenk et al. 1993; Przyklenk and Whittaker 2017). Repeated rIPC interventions (i.e. increasing the amount of rIPC applied) can improve cardiovascular parameters and clinical endpoints in healthy individuals and those with cardiovascular disease (CVD) risk factors, respectively. For example, repeated rIPC interventions ranging from 1 to 8 weeks have mediated improvements in vascular endothelial function in both healthy (Jones et al. 2014, 2015) and type 2 diabetic individuals (Maxwell et al. 2019), increases in coronary flow reserve in heart failure patients (Kono et al. 2014), increased presence of endothelial progenitor cells (Liang et al. 2015) and reduced diabetic foot ulcer wound size (Shaked et al. 2015). Collectively suggesting repeated rIPC interventions can improve cardiovascular function which may potentially translate to reduced risk of cardiovascular events and complications.

There is also emerging evidence that repeated rIPC interventions can have beneficial effects on the cerebrovasculature. A small number of studies have shown that repeated rIPC can increase cerebral blood flow (CBF) in stroke patients and importantly reduce the rate of stroke reoccurrence (Meng et al. 2012, 2015). Nevertheless, a recent study from our group, attempting to understand how rIPC exerts its effects on the cerebrovasculature, demonstrated that a single bout of rIPC has a negligible impact on CBF velocity (CBFv) or dynamic cerebral autoregulation (dCA) as a marker of cerebrovascular function (Carter et al. 2020). This observation may relate to the relatively small ‘dose’ of a single exposure to rIPC. Therefore, we examined the impact of 7-daily repeated rIPC intervention in individuals with type 2 diabetes, but found no impact on cerebrovascular function (Maxwell et al. 2019). A number of important methodological differences are evident between our recent studies (Carter et al. 2020; Maxwell et al. 2019) and the previous studies showing benefits on cerebrovascular health (Meng et al. 2012, 2015; Mi et al. 2016; Wang et al. 2017), primarily; participant population, length of rIPC intervention and measurement techniques. The length of the intervention and the number of rIPC bouts performed over the intervention, collectively the dose of rIPC is an important aspect to consider. It is possible that a larger dose of rIPC might have a greater effect on cerebrovascular function in healthy individuals or those with CVD risk factors. Therefore, by employing an 8-week intervention, which has improved peripheral vascular function (Jones et al. 2015), this may in turn result in cerebrovascular changes by increasing the “dose” of the rIPC from previous studies in our group (Maxwell et al. 2019).

An alternative approach to increase the preconditioning “dose” is to combine rIPC with exercise as a preconditioning stimulus. Exercise may potentially provide an additional, but not mutually exclusive, preconditioning stimulus for the vascular system (Thijssen et al. 2018). Accordingly, combining rIPC with exercise may increase the beneficial adaptations observed with rIPC alone. The mechanisms responsible for the beneficial effects of rIPC versus exercise preconditioning may differ. Where effects of rIPC may relate to both neural and humoral pathways (Anttila et al. 2016), benefits of exercise (preconditioning) may relate to repeated elevations in shear stress (Hambrecht et al. 2003; Thijssen et al. 2016; Tinken et al. 2010). Combining the repeated rIPC and exercise stimuli may complement each other, resulting in superior adaptation of peripheral and cerebral arteries.

Therefore, the primary aim of this study was to examine whether 8-week repeated rIPC can improve cerebrovascular and peripheral conduit artery function in individuals with increased risk of CVD, and whether these effects can be enhanced when combining rIPC with exercise training. It was hypothesised that exercise training would provide additional stimulus to elicit greater improvements in both cerebro- and peripheral vascular function compared to rIPC alone.

## Methods

### Participants

**Fig. 1:**
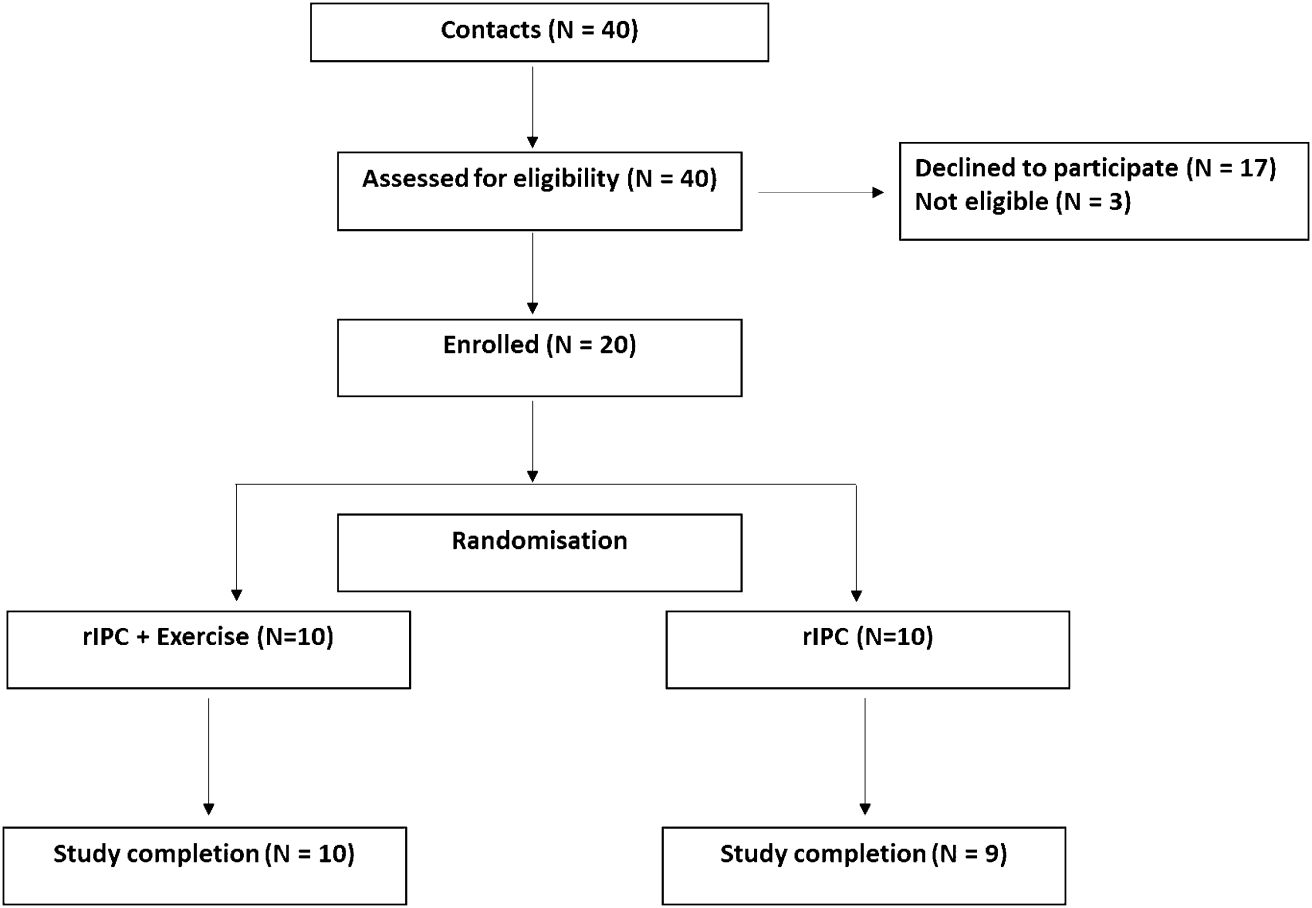
Screening, recruitment, retention and completion of the study

Nineteen participants with increased risk of CVD were recruited (Fig. 1). Participants were recruited on the criteria of: body mass index (BMI) > 30 kg/m^2^ or waist circumference ≥ 94 cm (male), ≥ 80 cm (female) as well as either raised blood pressure (> 130 /85 mmHg) or diagnosed with hypercholesterolemia and not currently undertaking any structured exercise programme. Raised blood pressure and hypercholesterolemia were confirmed based on medical history and current medications. Individuals were excluded if they had a history of stroke (including TIAs), myocardial infarction, thrombosis, congenital heart disease, type 1 diabetes or currently smoking. Participants were informed of the study protocol verbally and in writing before providing written informed consent. The study was approved by the local ethics committee (approval number 17/SPS/056) and conformed to standards set out by the *Declaration of Helsinki*. Registered clinical trial at ClinicalTrials.gov NCT03624452.

### Research design

Participants underwent two initial visits to the laboratory. Following an overnight fast and refraining from alcohol and exercise for 24 h and caffeine for 12 h, visit 1 consisted of anthropometric measurements, fasting blood glucose, assessment of cerebrovascular function and assessment of brachial artery endothelium function before and after a temporary ischaemia–reperfusion injury (IRI). Visit 2 consisted of a cardio-respiratory fitness test (*V*O_2peak_) to calculate exercise intensity. Both visits were conducted within four days of each other and all completed in a temperature-controlled laboratory (23 ± 1 °C). Participants were then randomly allocated into the rIPC + Exercise group (3 × rIPC and 3 × 50 min exercise per week) or rIPC group (3 × rIPC per week). All measurements performed in visits 1 and 2 were repeated within 7 days of end of the interventions for post measurements.

## Measurements

### Cerebral blood flow velocity and systemic haemodynamics

Following 20-min rest in the supine position, simultaneous middle cerebral artery (MCAv) and posterior cerebral artery velocities (PCAv) were continuously measured through the temporal window using transcranial Doppler ultrasonography (TCD) following standardised procedures (Willie et al. 2011). Two 2-MHz Doppler probes (Spencer Technologies, Seattle, USA) were adjusted until an optimal signal was identified and held in place using a Marc 600 head frame (Spencer Technologies, Seattle, USA). Participants were instrumented with a two-way valve mouthpiece (Hans Rudolph) from which end tidal CO_2_ (P_ET_CO_2_) was measured using a calibrated gas analyser (ML206 ADinstruments,Colorado Springs, USA). Continuous beat-by-beat blood pressure was obtained from a Finometer Pro (Finapres Amsterdam, Netherlands). Heart rate acquired from a three-lead electrocardiogram. All data were sampled at 50 Hz with the data acquisition system PowerLab via the interface LabChart 8 (ADinstruments, Colorado Springs, USA). An average of MCAv and PCAv were taken across the 5-min baseline period, and the same 5-min recording was used to characterise the relationship between MCAv and MAP during spontaneous fluctuations as well as the relationship between SBP and R–R intervals (baroreflex sensitivity).

### Squat–stand manoeuvres

Dynamic cerebral autoregulation (dCA) was assessed using a squat-to-stand procedure that induces transient changes in BP following previously stated guidelines (Claassen et al. 2016). Participants performed two sets of squat-to-stands at different frequencies; 0.10 Hz and 0.05 Hz for five minutes each, with five-minute rest in-between each set. During the manoeuvres, all participants were instructed to keep their eyes open and avoid strenuous breathing patterns (e.g., Valsalva manoeuver). The sequence in which the two frequency squat-stand manoeuvers were performed was randomised between participants. The same five-minute recordings were used to measure baroreflex sensitivity during forced oscillations in BP. Data were analysed in accordance with the most recent recommendations from the Cerebral Autoregulation Research Network (CARNet) using Transfer Function Analysis (Claassen et al. 2016). The cross spectrum between MCAv and MAP was determined for TFA by the MAP auto-spectrum to determine transfer function parameters absolute gain, normalised gain, phase and coherence at the point estimate of driven frequency. For spontaneous cerebral autoregulation, TFA gain, coherence and phase were band averaged across the very low (0.02–0.07 Hz), low (0.07–0.2 Hz) and higher frequency (0.2–0.4 Hz) ranges. TFA parameters were only included for subsequent analysis when coherence exceeded 0.4.

Baroreflex sensitivity data for both spontaneous and forced oscillations were analysed using a commercially available software Ensemble (Version 1.0.0.28, Elucimed, Wellington, New Zealand). Pressure-–cardiac interval transfer function was applied to the data to determine gain, phase and coherence. Similar to cerebral autoregulation data, spontaneous baroreflex were band averaged across the very low (0.02–0.07 Hz), low (0.07–0.2 Hz) and higher frequency (0.2–0.4 Hz) ranges, whilst forced oscillations were sampled at the point estimate of the driven frequency.

### Cerebrovascular reactivity to carbon dioxide

Following a 10-min rest period, a baseline measurement of CBFv, MAP and P_ET_CO_2_ was performed across 2 min, while participants breathed in room air before being switched to breathing directly from a Douglas bag (100 L) containing 5% CO_2_, 21% O_2_ and balanced nitrogen for a further 4 min. Cerebrovascular reactivity (CVR) was analysed by extracting a two-minute baseline average and an average of the last 30 s of the 4 min 5% CO_2_ breathing of CBFv, MAP, P_ET_CO_2_. Calculation of the CVR slopes was performed via linear regression analysis of the two time-points and are presented as CVR conductance indices (CbVCi), CVR to carbon dioxide (CVR_CO2_) and MAP reactivity (Barnes et al. 2018; Miller et al. 2018). CbVCi was calculated as MCAv/MAP.

### Brachial artery endothelium-dependent vasodilation

Brachial artery endothelial function was assessed using the flow-mediated dilation (FMD) technique following 20 min of supine rest (Thijssen et al. 2019b). Images of the left brachial artery were acquired using high-resolution ultrasound (T3300; Terason, Burlington, MA). Diameter, flow and shear stress were measured prior to and following 5 min of forearm cuff inflation (D.E. Hokanson, Bellevue, WA). All FMD measurements were performed by the same sonographer with a day-to-day coefficient of variation in FMD% of 11% and a coefficient of variation of 3% for baseline artery diameter which is deemed good–excellent based on previous analysis (van Mil et al. 2016).

Analysis was performed using custom-designed edge-detection and wall-tracking software, which is largely independent of investigator bias. Previous articles contain detailed descriptions of our analytical approach (Black et al. 2008; Woodman et al. 2001). Reproducibility of diameter measurements using this semi-automated software is significantly better than manual methods, significantly reduces observer error, and possesses within-day coefficient of variation of 6.7% (Woodman et al. 2001). Allometric scaling for baseline diameter was performed (Atkinson and Batterham 2013). FMD analysis was performed by a researcher blinded to the group allocation using a single-blinded coding-randomised procedure.

### Ischaemia–reperfusion injury

The rapid inflation/deflation pneumatic cuff was positioned proximally around the upper arm to provide occlusion for 15 min. This ensured the brachial artery was in the ischaemic zone and exposed to IRI. The cuff was inflated for 15 min and was followed by a 15-min reperfusion (cuff deflation) phase before a second FMD was performed to assess post IRI FMD. This is a safe and frequently used method utilised to assess IRI-induced endothelial dysfunction (Aboo Bakkar et al. 2018, Carter et al. 2014, Loukogeorgakis et al. 2010, Thijssen et al. 2019a, van den Munckhof et al. 2013).

### Maximal oxygen uptake

The cardio-respiratory fitness test (*V*O_2peak_) was performed on a treadmill (H/P Cosmos, Pulsar 4.0, Nussdorf-Traunstein, Germany) to quantify peak aerobic capacity. A modified version of the Bruce et al. (1973)* protocol* was adopted as this is frequently used protocol in sedentary/high-risk populations (Pugh et al. 2013; Sprung et al. 2013). Following a 5-min warm-up period at a self-selected speed, the protocol begins with a 2-min stage at 2.2 km/h on a flat gradient, followed by 2 min at 2.7 km/h at a 5% gradient. Subsequently, stepwise increments in speed and gradient are applied every minute until volitional exhaustion. Breath-by-breath expired gases were continuously monitored (Oxycon Pro, Jaeger, Hochberg Germany) for oxygen consumption (ml/kg^/^min) and were averaged over 15 s. Peak oxygen uptake was calculated from the highest consecutive 15-s period of expired gas fractions. Heart rate was measured continuously using short-range telemetry (RS800, Polar, Finland) alongside subjective effort (RPE) using the 6–20 Borg scale. All participants reached the criteria for volitional exhaustion based upon heart rate, Borg scale and respiratory exchange ratio.

### Fasting blood glucose

Blood samples were obtained from the antecubital vein via standard venepuncture technique (Vacutainers Systems, Becton–Dickinson). All samples were collected into vacutainers containing a polymer gel for serum separation. Centrifugation for 10 min at 1000 *g* at 4 °C was applied and samples were stored at − 80 °C for subsequent analysis. Plasma glucose was determined spectrophotometrically using commercially available kits (Randox Laboratories, Antrim, UK) with each sample analysed in duplicate.

### Remote ischaemic preconditioning

All participants in both groups performed three bouts of rIPC per week for 8 weeks. A single bout of rIPC consisted of a pressure cuff (Welch Allyn DuraShock™ DS45, New York, USA) inflated around the upper arm (220 mmHg) for five minutes preceded by five minutes of reperfusion, repeated four times (total time 40 min). The arm in which the rIPC was applied was randomised and participants were free to perform the rIPC bouts freely and not follow a pre-set routine. All participants were provided with an intervention diary to increase compliance which was 100%.

### Exercise intervention

Those randomly assigned to the rIPC + Exercise group performed three 50-min exercise sessions per week for 8 weeks (98% compliance). All sessions were performed on a cycle ergometer (Wattbike Trainer, Wattbike Limited, country) in a temperature-controlled laboratory (18 ± 2 °C). The intensity of the exercise sessions was set to 70% maximum heart rate (HR_max_) which was calculated directly from the pre-intervention *V*O_2peak_ assessment, and all sessions were supervised in order to ensure target HR was achieved and maintained throughout the session. This exercise intervention met the United Kingdom government most recent criteria of 150 min of moderate exercise per week (Gibson-Moore 2019) and was set at an intensity which previous research shows is both feasible and effective at improving cardio-respiratory fitness and vascular endothelial function (Bailey et al. 2016).

### Statistical analysis

Analysis was performed using Statistical Package for Social Sciences (Version 26; SPSS Inc., Chicago, IL). Baseline characteristics between conditions (Table 1) were analysed using an independent samples t-test. All other data were analysed using a linear mixed model, with delta changes (**∆)** from week 0 to week 8 calculated and added to the model as a dependent variable and pre-intervention data used as a covariate. Statistical significance was delimited at *P* < 0.05 and exact *P* values are cited (*P* values of ‘0.000’ provided by the statistics package are reported as < 0.001). Significant interactions and main effects were followed up using LSD pairwise comparison. Data are presented as mean and 95% confidence intervals. Our primary outcome variable for the study was LF gain at 0.10 Hz. No studies to date have examined LF gain with rIPC over an 8-week intervention period. We there based our sample size on studies that have investigated rIPC or exercise over 8 weeks or more that had primary outcome variables on cerebrovascular reactivity to CO_2_ and FMD in healthy young and older individuals without overt cardio, cerebro or respiratory disease (Jones et al. 2015; Murrell et al. 2013).

**Table 1:** Baseline characterises and medications of both groups

Baseline characteristics	rIPC + exercise (4 females and 6 males)	rIPC only (2 females and 7 males)	*P* value
Age (years)	52 ± 8	51 ± 12	0.87
Height (m)	1.69 ± 0.10	1.74 ± 0.10	0.26
Weight (kg)	97.8 ± 21.5	108.2 ± 21.7	0.31
Body mass index (kg/m^2^)	34 ± 5	35 ± 5	0.56
Waist circumference (cm)	107 ± 17	109 ± 13	0.75
Resting heart rate (bpm)	69 ± 8	69 ± 6	0.96
Systolic blood pressure (mmHg)	137 ± 15	139 ± 12	0.75
Diastolic blood pressure (mmHg)	80 ± 13	84 ± 6	0.42
Mean arterial blood pressure (mmHg)	99 ± 12	102 ± 5	0.50
Fasting blood glucose (mmol/L)	5.9 ± 0.7	6.0 ± 0.7	0.70
Medications			
Statins	3 (30%)	3 (33%)	
β-blockers	1 (10%)	1 (11%)	
Calcium channel blockers	1 (10%)	0 (0%)	
Alpha-1 adrenergic blockers	1 (10%)	0 (0%)	
Angiotensin-converting-enzyme inhibitors	3 (30%)	2(22%)	
Biguanides	2 (20%)	2 (22%)	

## Results

### Resting haemodynamics

The directional changes in MAP (*P* = 0.45), MCAv (*P* = 0.30) and PCAv (*P* = 0.56) were negligible between interventions (Table 2).

**Table 2:** Baseline haemodynamic, cardio-respiratory fitness and fasting blood glucose data from before (week 0) and after (week 8) each intervention

	rIPC + Exercise		rIPC only		LMM
	Week 0	Week 8	Week 0	Week 8	Condition
MCAv (cm s^−1^)	56 ± 10	57 ± 9	54 ± 14	53 ± 12	0.30
PCAv (cm s^−1^)	37 ± 3	36 ± 4	36 ± 3	36 ± 2	0.83
P_et_CO_2_ (mmHg)	38.5 ± 3.1	38.0 ± 3.7	41.6 ± 2.7	39.6 ± 3.5	0.35
MAP (mmHg)	99 ± 13	95 ± 11	102 ± 7	99 ± 5	0.45
*V*O_2peak_ (ml/kg/min)	22.9 ± 5.4	25.7 ± 6.2	23.5 ± 2.9	23.6 ± 2.8	0.69
Fasting blood glucose (mmol/L)	5.9 ± 0.7	5.9 ± 0.7	6.4 ± 1.6	6.5 ± 1.4	0.27

Data presented as means ± SD

*MCAv* middle cerebral artery velocity, *PCAv* posterior cerebral artery velocity, *P*_*et*_*CO*_*2*_ partial pressure of end tidal carbon dioxide, *MAP* mean arterial pressure, *rIPC* remote ischaemic preconditioning, *LMM* linear mixed model

### Cerebrovascular function

There was no difference in any parameter of dCA between interventions at rest (*P* > 0.05, Table 3) or during squats–stand manoeuvres (*P* > 0.05, Table 4). There was no difference between interventions on CVR_CO2_, CbVCi_Co2_ or MAP reactivity_Co2_ (*P* > 0.05, Table 5).

**Table 3:** Power spectral and transfer function analysis of dynamic cerebral autoregulation during spontaneous changes in BP and CBFv

	rIPC + exercise		rIPC only		LMM
	Week 0	Week 8	Week 0	Week 8	
Power spectrum					
Baseline MCAv power (cm/s^2^)					
VLF	2.69 ± 1.17	2.86 ± 1.53	2.41 ± 2.15	2.87 ± 1.37	0.13
LF	1.69 ± 0.96	1.81 ± 1.30	1.58 ± 0.122	2.61 ± .1.86	0.84
HF	0.47 ± 0.24	0.52 ± 0.28	0.81 ± 0.27	0.97 ± 0.21	0.19
Baseline BP power (mmHg^2^)					
VLF	4.23 ± 1.47	3.43 ± 1.95	4.52 ± 2.72	4.96 ± 3.62	0.21
LF	2.26 ± 1.43	2.28 ± 2.00	2.05 ± 1.17	1.77 ± 0.86	0.32
HF	0.44 ± 0.13	0.40 ± 0.22	0.44 ± 0.31	0.43 ± 0.23	0.69
Transfer function					
Spontaneous oscillations					
VLF gain (cm s mmHg)	0.68 ± 0.23	0.73 ± 0.32	0.51 ± 0.08	0.51 ± 0.06	0.72
VLF Ngain (%/mmHg)	1.15 ± 0.22	1.34 ± 0.62	0.98 ± 0.25	1.35 ± 0.75	0.69
VLF phase (radians)	0.77 ± 0.32	0.87 ± 0.41	1.03 ± 0.48	1.08 ± 0.69	0.98
VLF coherence	0.54 ± 0.14	0.51 ± 0.01	0.52 ± 0.23	0.50 ± 0.20	0.14
LF gain	0.74 ± 0.29	0.75 ± 0.29	0.75 ± 0.25	0.88 ± 0.34	0.16
LF Ngain	1.26 ± 0.41	1.40 ± 0.41	1.35 ± 0.45	1.43 ± 0.59	0.54
LF phase	0.52 ± 0.38	0.65 ± 0.31	0.89 ± 0.49	0.74 ± 0.41	0.31
LF coherence	0.56 ± 0.09	0.55 ± 0.12	0.50 ± 0.11	0.51 ± 0.21	0.31
HF gain	1.00 ± 0.43	0.99 ± 0.50	0.97 ± 0.53	1.00 ± 0.31	0.20
HF Ngain	1.77 ± 0.42	1.81 ± 0.70	1.47 ± 0.32	1.84 ± 0.45	0.43
HF phase	0.14 ± 0.66	0.09 ± 0.51	0.33 ± 0.21	0.18 ± 0.08	030
HF coherence	0.61 ± 0.08	0.63 ± 0.08	0.63 ± 0.06	0.63 ± 0.07	0.75

Data presented as means ± SD

*PS* power spectrum, *VLF* very low frequency, *LF* low frequency, *HF* high frequency, *MCAv* middle artery cerebral velocity, *BP* blood pressure, *Ngain* normalised gain, *LMM* linear mixed model

**Table 4:** Power spectrum densities and transfer function of forced oscillations in mean arterial pressure and cerebral blood flow velocity during squat–stand manoeuvres

	rIPC + exercise		rIPC only		LMM
	Week 0	Week 8	Week 0	Week 8	Condition
0.05 Hz					
Power spectrum					
BP power (mmHg^2^)	69.7 ± 31.7	78.4 ± 43.2	73.3 ± 32.4	78.2 ± 42.9	0.86
MCAv power (cm/s^2^)	40.1 ± 25.5	42.2 ± 24.6	35.1 ± 31.43	32.3 ± 26.6	0.19
Transfer function					
Gain (cm s mmHg)	0.51 ± 0.15	0.49 ± 0.15	0.52 ± 0.10	0.50 ± 0.12	0.94
Ngain (%.mmHg^−1^)	0.99 ± 0.36	0.97 ± 0.23	0.99 ± 0.16	1.01 ± 0.08	0.80
Phase (radians)	0.94 ± 0.47	0.97 ± 0.36	1.01 ± 0.30	1.01 ± 0.08	0.48
Coherence	0.92 ± 0.05	0.91 ± 0.06	0.82 ± 0.09	0.85 ± 0.10	0.50
0.10 Hz					
Power spectrum					
BP power (mmHg^2^)	111 ± 79.5	113 ± 87	116 ± 108	111 ± 99	0.91
MCAv power (cm/s^2^)	43.1 ± 36	40.3 ± 33	45.6 ± 29	41.5 ± 32	0.98
Transfer function					
Gain (cm s mmHg)	0.66 ± 0.15	0.68 ± 0.13	0.62 ± 0.24	0.63 ± 0.20	0.85
Ngain (%.mmHg^−1^)	1.18 ± 0.30	1.34 ± 0.23	1.25 ± 0.29	1.23 ± 0.17	0.40
Phase (radians)	0.40 ± 0.14	0.42 ± 0.19	0.49 ± 0.17	0.46 ± 0.18	0.52
Coherence	0.72 ± 0.07	0.72 ± 0.08	0.72 ± 0.04	0.71 ± 0.08	0.91

Data presented as means ± SD

*rIPC* remote ischemic preconditioning, *BP* blood pressure, *MCAv* middle cerebral artery velocity, *LMM* linear mixed model

**Table 5:** Cerebral reactivity to 5% carbon dioxide

	rIPC + Exercise		rIPC only		LMM
	Week 0	Week 8	Week 0	Week 8	Condition
CVRCO2 (cm s/mmHg)	1.85 ± 0.90	1.90 ± 0.59	1.65 ± 0.70	1.58 ± 0.69	0.41
CbVCiCo2 (cm s/mmHg^2^)	0.018 ± 0.011	0.013 ± 0.005	0.009 ± 0.006	0.017 ± 0.011	0.26
MAP reactivityCo2 (mmHg/mmHg)	0.5 ± 0.4	0.9 ± 0.6	1.1 ± 0.7	1.5 ± 0.7	0.37

Data presented as means ± SD

*CVR*_*Co2*_ cerebral reactivity to carbon dioxide, *CvVCi*_*Co2*_ cerebrovascular conductance index to carbon dioxide, *MAP reactivity*_*Co2*_ mean arterial pressure reactivity to carbon dioxide, *rIPC* remote ischemic preconditioning, *LMM* linear mixed model

### Brachial artery endothelium-dependent vasodilation

**Fig. 2:**
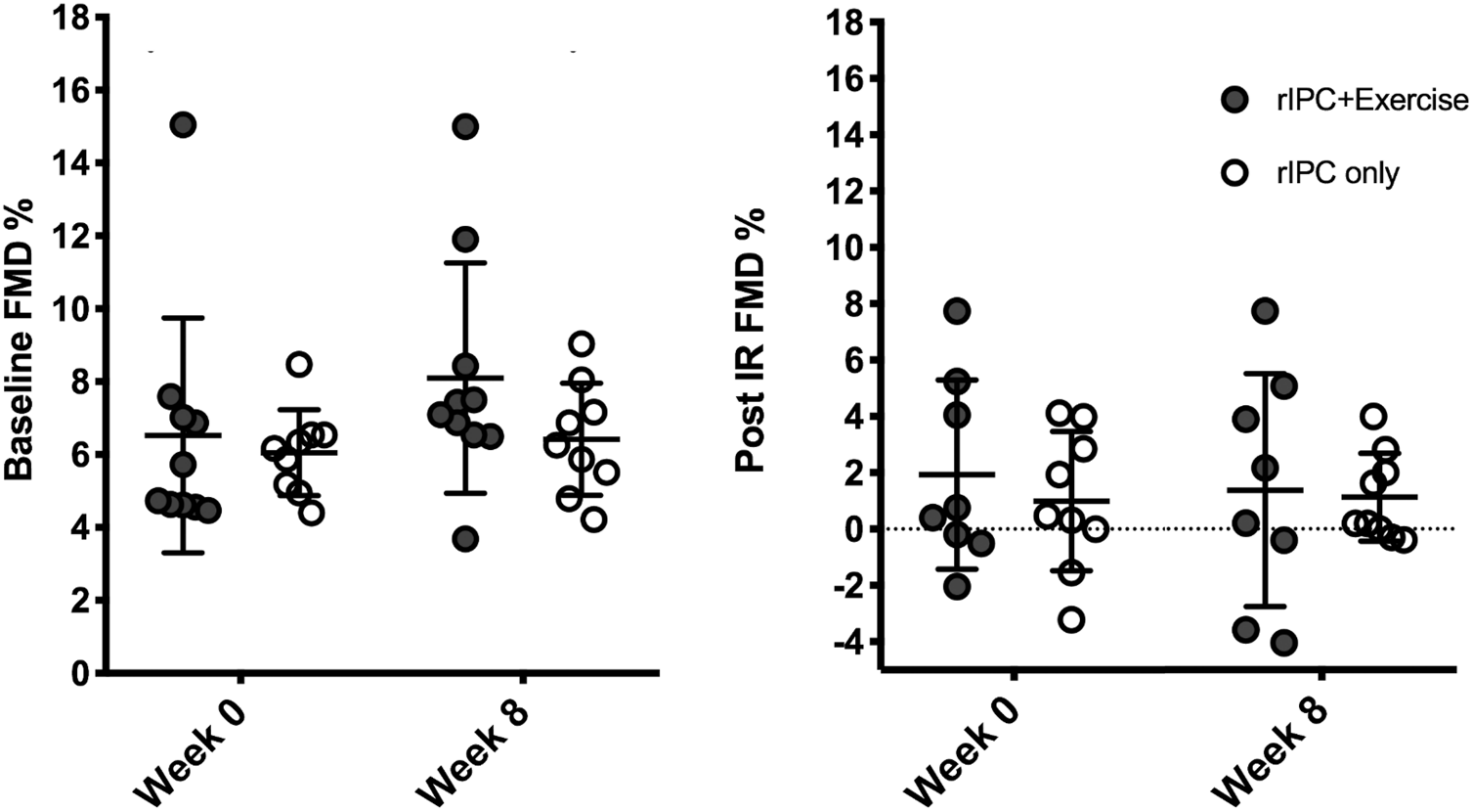
Individual data points with means ± SD for baseline flow-mediated dilation (FMD, left panel) and post ischaemic–reperfusion (IR) injury flow-mediated dilation (right panel)

Brachial artery FMD% increased from week 0 to week 8 by 1.6% (95% CI; 0.4, 2.8) in the rIPC + Ex intervention and by 0.3% (95%; − 1.1, 1.5) in rIPC only intervention, there was no statistical difference between interventions (*P* = 0.65, Table 6). Post IR injury FMD% reduced by 0.5% (95%; − 2.2, 1.3) in rIPC + EX and by 0.2% (95%; − 1.7, 1.6) in the rIPC only from week 0 to week 8. The IR injury resulted in an overall significant decrease in FMD% (5.1%, (95%: 3.7, 6.4, *P* < 0.001, Fig. 2). The directional changes in brachial artery FMD following the IR injury in both interventions were not different (*P* = 0.50, Fig. 2).

**Table 6:** Brachial artery characteristics before and after an ischaemia–reperfusion injury

	rIPC + exercise		rIPC only		LMM
	Week 0	Week 8	Week 0	Week 8	Condition
Baseline					
Resting diameter (mm)	4.0 ± 0.9	4.1 ± 1.0	4.2 ± 0.7	4.1 ± 0.8	0.40
FMD%	6.5 ± 3.2	8.1 ± 3.2	6.1 ± 1.2	6.4 ± 1.6	0.65
Scaled FMD%	6.5 ± 2.6	7.9 ± 1.3	6.1 ± 2.4	6.3 ± 2.3	0.35
Time to peak (s)	77 ± 32	67 ± 29	72 ± 24	63 ± 26	0.83
Shear AUC (103)	28.2 ± 15.0	23.3 ± 14.8	22.2 ± 14.9	22.0 ± 14.2	0.64
Post ischaemia–reperfusion injury					
Resting diameter (mm)	4.4 ± 1.0	4.4 ± 1.0	4.3 ± 0.7	4.6 ± 0.9	0.95
FMD%	1.9 ± 3.3	1.4 ± 4.1	1.0 ± 2.5	0.8 ± 1.2	0.50
Scaled FMD%	1.9 ± 2.9	1.3 ± 3.7	1.0 ± 3.0	1.0 ± 3.0	0.56
Time to peak (s)	79 ± 35	73 ± 32	63 ± 39	62 ± 33	0.87
Shear AUC (103)	21.2 ± 7.1	19.0 ± 5.7	13.8 ± 10.4	12.1 ± 7.1	0.24

Data presented as means ± SD

*FMD* flow-mediated dilation, *AUC* area under the curve, *LMM* linear mixed model

No difference between interventions were evident in any other characteristics in resting brachial artery FMD and post IR injury FMD (*P* > 0.05, Table 6, respectively).

### Cardiorespiratory fitness

*V*O_2peak_ increased by 2.8 ml/kg/min (95%; 1.7, 3.9) following the rIPC + Ex intervention and increased by 0.1 ml/kg/min (95%; − 1.0, 1.4) following the rIPC only intervention. There was no statistical difference between interventions (*P* = 0.69, Table 2).

### Blood glucose

There was no statistical difference between interventions (*P* = 0.27, Table 2).

## Discussion

The present study aimed to examine whether increasing the rIPC dose by combining rIPC with aerobic exercise training resulted in improvements in the cerebral and peripheral vasculature in individuals with increased risk of CVD. The results of this study suggest that increasing the preconditioning stimulus by combining rIPC intervention with exercise training does not mediate greater changes in the cerebral or peripheral vasculature when compared directly to rIPC alone.

The data from this study show that combining exercise training with rIPC mediates a 1.6% increase in FMD. Given that a 1% increase in FMD is indicative of a 8–13% reduced risk of cardiovascular events (Thijssen et al. 2019b), the increase observed following the rIPC combined with exercise in the current study is of clinical importance. Intriguingly, a 0.3% increase in FMD was observed in the rIPC only group. This is somewhat smaller than a previous study employing the same repeated rIPC intervention in young healthy individuals (Jones et al. 2015). The differences in responses to the repeated rIPC intervention between the two studies may be explained by differences in participant groups. Indeed, there is evidence to suggest differences in the time course of changes in FMD are apparent depending on health status following exercise interventions (Schreuder et al. 2015). Similarly, an acute bout of rIPC has attenuated efficacy in older and diseased individuals in response to FMD following an endothelial reperfusion injury (Seeger et al. 2016, van den Munckhof et al. 2013). Collectively, the data presented in this study suggest that either; (i) rIPC, with or without exercise is unable to evoke improvements in cerebral and peripheral vascular function or (ii) individuals at risk of CVD may require large ‘doses’ of rIPC and exercise to mediate improvements compared to a healthier population.

Despite increasing the dose of rIPC (by adding exercise training), we observed negligible changes in all the markers of cerebrovascular function including CBFv with either intervention. The repeated rIPC interventions that have shown increases in CBF and CBFv were evident following much longer interventions and in patient groups with overt cerebrovascular disease (Meng et al. 2012, 2015; Wang et al. 2017). Nevertheless, the aforementioned studies have not examined cerebrovascular function to understand how the positive changes in CBF and risk of stroke occur. The markers of cerebrovascular function dCA and CVR are recognised as an independent predictor of ischaemic stroke (Markus and Cullinane 2001) and provide in-depth information about mechanoreceptor and chemoreceptor control, respectively (Hoiland et al. 2019; Rubanyi et al. 1990). Given the impact of repeated rIPC in stroke patients, we anticipated our repeated rIPC intervention to mediate positive effects on the cerebral vasculature. Moreover, we expected that the addition of exercise would mediate a greater response, given that some (Akazawa et al. 2012; Alfini et al. 2019; Ivey et al. 2011; Murrell et al. 2013; Zhu et al. 2011), but not all (Drapeau et al. 2019; Lewis et al. 2019; Tanne et al. 2005) previous exercise training studies have shown improvements in CVR, dCA and CBFv with exercise training interventions in a number of ages and disease groups. Whilst we cannot discount that a longer intervention may have mediated positive changes, or that rIPC and exercise might have had an impact on cerebrovascular function in individuals with overt disease, our data suggest that repeated rIPC intervention has negligible impact on cerebrovascular function.

Numerous studies have acknowledged that rIPC provides protection against the IR injuries, including the same IR model adopted in the current study by means of attenuated differences between pre and post IR injury FMD’s (Luca et al. 2013, Maxwell et al. 2019, van den Munckhof et al. 2013). Interestingly, no protective effect from either intervention on IR injury FMD was observed. This finding may be attributed to rIPC intervention intensity, given that those aforementioned studies applied either acute (one bout) or daily rIPC in the lead up to the IR injury, building a more substantial preconditioning effect. Additionally, and again somewhat surprising was the inability of the exercise to elicit any protection, especially given the suggestion that exercise does pose a preconditioning-like effect (Thijssen et al. 2018). Both lifelong exercise (Maessen et al. 2017) and short-term exercise interventions (Thijssen et al. 2019a) have resulted in increased tolerance to endothelial IR injury. Given a clinically important change in fitness was observed in the rIPC combined with exercise intervention, it might be more plausible that exercise type may represent an explanation for differences in post IR injury results. However, Thijssen et al. (2019a) found that the protective effects were present and not different following moderate- and high-intensity exercise, yet mode and intensity of exercise may still play a major role in FMD, both pre and/or post IR. This present study implemented a primarily lower body (cycling) exercise intervention, whereby the shear stress stimulus is going to be greater in blood vessels of the legs; nevertheless, it is important to recognise that even during lower limb exercise (cycling), increases in shear stress do occur in the brachial artery (Green et al. 2017)—the site in which endothelial function was assessed in this study. Indeed the shear stress stimulus represents a major contributor to changes in vascular function, especially given evidence showing cycling training improves brachial FMD, but not when the blood flow response in occluded (Birk et al. 2012). Interestingly, in a population of obese individuals, a moderate intensity exercise intervention, similar to that implemented in this study did not increase brachial FMD; whereas, a high intensity interval intervention did improve endothelial function (Sawyer et al. 2016). One study employing an identical exercise intervention in terms of type, duration and intensity to this present study observed significant increases in brachial FMD albeit in healthy post-menopausal women (Bailey et al. 2016), yet as a result of the addition of rIPC into the exercise intervention in the study, direct comparisons are challenging. Indeed, we cannot categorically state whether a different exercise modality would have mediated different cardiovascular responses. Cycling was selected as the exercise type not only based on the aforementioned studies (Bailey et al. 2016; Sawyer et al. 2016), but also because cycling offers an accessible modality for those with functional limitations and reduces the risk of musculoskeletal injuries. Meta-analysis suggests that all forms of aerobic training are effective at improving cardiovascular parameters such as endothelial function, with exercise intensity rather than exercise modality being the most prominent factor (Ashor et al. 2015).

Assessing further vascular parameters may provide additional information in understanding the effects of an IR injury, with some studies measuring low flow-mediated constriction in order to explore a different mechanistic view (Carter et al. 2014; Rakobowchuk et al. 2013). Alternatively, methodological differences may explain differences in post IR injury FMD results, with the present study adopting a 15-min-ischaemia–15 min-reperfusion model; whereas, Thijssen et al. (2019a) used 5 min of ischaemic handgrip exercise followed by 15-min reperfusion. Further research is warranted to understand the potential preconditioning effect of exercise across a wider population.

We acknowledge a number of considerations related to measurement techniques and study design which are noteworthy. The use of TCD assesses blood velocity rather than blood flow as arterial diameter is not taken into consideration. However, evidence does show that MCAv is a reliable index of cerebral blood flow if the insonated vessel maintains a constant diameter across experimental conditions. MCA diameter has been shown to be consistent during modest changes in CO_2_ (± 5 mmHg) (Ainslie and Hoiland 2014). The model employed to induce a temporary endothelial IR injury is used only as a surrogate index to cardiac tissue. Nonetheless, this is a frequently used model (Thijssen et al. 2019a, van den Munckhof et al. 2013) and applying this technique significantly decreases plasma nitrite and plasma nitrate concentrations, indicating a reduction in NO bioavailability (Aboo Bakkar et al. 2018). Our study population is limited to individuals with risk factors for CVD rather than in those with overt CVD; therefore, whether the response to rIPC with or without exercise mediates a different response certainly warrants future investigation. Furthermore, the participants recruited within both groups of the present study were prescribed various different antihypertensive medications (Table 1). Nevertheless, given the small differences and the lack of significant effects in the primary variable we believe that this had negligible impact of the study findings. Finally, our research design did not include an exercise only condition; therefore, within the rIPC + Ex intervention, we cannot precisely determine whether changes in FMD were attributed more towards the exercise or rIPC. Therefore future studies should consider incorporating an additional exercise only interventions to provide clarity.

In conclusion, combining 8 weeks of rIPC with exercise does not result in greater changes in cerebrovascular function and/or peripheral endothelial function compared to a repeated rIPC-only intervention in individuals at an increased risk of CVD. Therefore, based on these data, careful consideration and further investigation are recommended to examine whether rIPC offers a beneficial short-term intervention for improvement of systemic vascular health in at-risk individuals.

## Abbreviations

BRS: Baroreflex sensitivity
CBFv: Cerebral blood flow velocity
CVD: Cardiovascular disease
CVR: Cerebrovascular reactivity
dCA: Dynamic cerebral autoregulation
Ex: Exercise
FMD: Flow-mediated dilation
IR: Ischaemia–reperfusion
MAP: Mean arterial pressure
MCAv: Middle cerebral artery velocity
P_ET_CO_2_: Partial pressure of carbon dioxide
rIPC: Remote ischaemic preconditioning
TCD: Transcranial Doppler
TFA: Transfer function analysis
